# Risk factors for mortality in the late amputation of necrotizing fasciitis: a retrospective study

**DOI:** 10.1186/s13017-018-0207-0

**Published:** 2018-10-01

**Authors:** Chia-Peng Chang, Cheng-Ting Hsiao, Chun-Nan Lin, Wen-Chih Fann

**Affiliations:** 1 Department of Emergency Medicine, Chang Gung Memorial Hospital, No.6, Sec. W., Jiapu Rd, Puzi City, Chiayi County 613 Taiwan; 2 Department of Medicine, Chang Gung University, Taoyuan, Taiwan

**Keywords:** Necrotizing fasciitis, Amputation, Soft tissue infection, Risk factor, LRINEC

## Abstract

**Background:**

Necrotizing fasciitis (NF) is a rapidly progressive infectious disease that primarily involves the fascia and subcutaneous tissue. If not promptly treated, it can lead to morbidity as well as mortality. It can affect any part of the body, most commonly the extremities. Early and aggressive surgical treatment is the proper way of management. The purpose of this study was to identify the risk factors for mortality in late amputation among NF patients that may be used in routine clinical practice to prevent mortality.

**Methods:**

A retrospective cohort study of hospitalized patients with NF was conducted in a tertiary teaching hospital in Taiwan between March 2015 and March 2018. All collected data were statistically analyzed.

**Results:**

A total of 582 patients with NF were included; 35 of them had undergone amputation (7 primary and 28 late amputations), with a 6% amputation rate. Thirteen amputated patients still died eventually (all in the late amputation group). Significant risk factors for mortality identified in the late amputation group included hemorrhagic bullae (*p* = 0.001, OR 4.7, 95% confidence interval (CI) 2.68–8.69), peripheral vascular disease (*p* < 0.001, OR 3.2, 95% CI 1.12–10.58), bacteremia (*p* = 0.021, OR 2.87, 95% CI 2.07–5.96), and Laboratory Risk Indicator of Necrotizing Fasciitis (LRINEC) score > 8 (*p* < 0.001, OR 1.97, 95% CI 1.28–4.61). *Vibrio vulnificus* was the main causative organism based on our study, but the microbiology results showed no significant correlation.

**Conclusion:**

NF patients with hemorrhagic bullae, comorbidity with peripheral vascular disease, presence of bacteremia, or LRINEC score > 8 should receive early and primary amputation in order to prevent mortality.

## Background

Necrotizing fasciitis (NF) is a serious form of infection involving rapidly spreading inflammation and extensive necrosis of the skin, subcutaneous tissue, and superficial fascia [1]. In order to successfully treat NF, two important factors must always be present: awareness of the disease, in spite of its rare occurrence, and consequently immediate therapy (surgical and antibiotic treatment). The treatment of choice for NF is rapid surgical debridement and broad-spectrum antibiotic therapy [2]. Delayed treatment may result in extensive loss of soft tissue associated with limb loss; moreover, the risk of mortality is also increased. Even with aggressive treatment, patients may suffer significant morbidity such as amputation and organ failure [3–5]. Various predictors of mortality based on predisposing factors have been reported by different investigators. Previous studies have identified advanced age (> 60 years), *Aeromonas* and *Vibrio* infection, liver cirrhosis, cancer, hypotension, band polymorphonuclear neutrophils > 10%, and serum creatinine level > 2 mg/dL to be independent predictors of mortality in NF cases [6, 7]. Khamnuan et al. identified that clinical predictors for amputation in patients with NF included diabetes mellitus, soft tissue swelling, skin necrosis, gangrene, and serum creatinine values > 1.6 mg/dL on admission [8]. Lee et al. reported that treatment delayed beyond 3 days was an independent factor indicating a poor prognosis in *Vibrio* NF [9]*.* However, limited data exist with regard to with mortality and delayed amputation. This study investigated factors associated with mortality in late amputation among NF patients that could be used in clinical practice.

## Methods

The institutional review board of our hospital approved this retrospective study. In all, 582 patients were enrolled based on two criteria: (1) surgically proven diagnosis of NF and (2) treatment received between March 2015 and March 2018. Thirty-five patients underwent amputation during hospitalization, of which 7 were amputated within 3 days of admission, defined as primary amputation, while the other 28 were amputated beyond 3 days, defined as late amputation. Thirteen amputated NF patients died during hospitalization. We defined the variables as follows: wound, the presence of any wound on the affected limb, toe, or finger; hemorrhagic bullae, confirmed as hemorrhagic bullae by the emergency physician in the medical records and clinical photos. Comorbidities and admission laboratory data were extracted through chart review. All patients were assessed by emergency physicians as soon as they were admitted. They received broad-spectrum antibiotic treatment for anaerobic and aerobic bacteria as well as early surgical debridement including fasciotomy or primary amputation post-diagnosis. Each patient’s medical record was screened for documentation of NF to confirm the diagnosis. Those with NF involving the head, neck, or trunk were excluded. Amputation sites included the fingers, toes, hands, forearms, and below and above the knee. Baseline demographic characteristics, laboratory findings, and clinical presentation were compared between mortality in the late amputation and survival of the amputation groups.

### Statistical analysis

All data were analyzed using SPSS Statistics version 20.0 (IBM Corp., Armonk, NY, USA). Continuous variables were analyzed using the *t* test, and categorical variables were analyzed using the chi-square test, except where 20% of cells had expected counts of < 5, in which case Fisher’s exact test was used. Multivariate logistic regression analysis was performed to identify the predictors of mortality in the late amputation NF cohort along with the odds ratio (OR) and 95% confidence interval (CI). *p* values less than 0.05 were considered to be statistically significant.

## Results

From a total number of 582 patients, 35 were amputated resulting in an amputation rate of 6%. Among 35 amputated patients, 7 were categorized as primary, while 28 were categorized as late amputations. Thirteen amputated patients died during hospitalization, who all belonged to the late amputation group, and the other 22 patients survived. As shown in Table 1, in the analysis of clinical condition, comorbidity, and laboratory evaluations, there were statistically significant differences with hemorrhagic bullae (53.8% vs. 15.4%; *p* = 0.001), diabetes mellitus (76.9% vs. 41.4%; *p* = 0.004), peripheral artery disease (23.1% vs. 1.2%; *p <* 0.001), C-reactive protein level (191.9 mg/dL vs. 134.1 mg/dL), bacteremia (69.2% vs. 36.0%; *p* = 0.001), and Laboratory Risk Indicator for Necrotizing Fasciitis (LRINEC) score > 8 (61.5% vs. 36.7%; *p <* 0.001) between mortality in the late amputation and survival in the amputation groups. All significant variables (with *p <* 0.05) were included in the multivariable logistic regression analysis. The multivariate logistic regression analysis revealed that independent risk factors for mortality in late amputation included hemorrhagic bullae (*p* = 0.001, OR 4.75, 95% CI 2.68–8.69), peripheral vascular disease (*p* < 0.001, OR 3.2, 95% CI 1.12–10.58), LRINEC score > 8, (*p* < 0.001, OR 1.97, 95% CI 1.28–4.61), and the presence of bacteremia (*p* = 0.021, OR 2.87, 95% CI 2.07–5.96) (Table 2).

**Table 1:** Clinical characteristics between mortality in the late amputation and survival in the amputation group

Variable	Mortality in late amputation (*n* = 13)		Survival in amputation (*n* = 22)		*p* value
Mean age (years)	65.08	± 10.94	66.35	± 14.76	0.762
Clinical condition, no.(%)					
Wound	9	(69.2%)	9	(42.0%)	0.059
Hemorrhagic bullae	7	(53.8%)	13	(35.4%)	0.001^*^
Comorbidity, no. (%)					
Diabetes mellitus	10	(76.9%)	8	(36.4%)	0.014^*^
CKD	5	(38.5%)	10	(45.5%)	0.286
Liver cirrhosis	1	(7.7%)	5	(22.7%)	0.327
PAD	5	(38.5%)	2	(9.1%)	< 0.001^*^
Admission data					
WBC count (× 10^3^/μL)	13.9	(± 6.5)	14.8	(± 7.8)	0.693
CRP (mg/dL)	191.9	(± 103.3)	134.1	(± 111.7)	0.012^*^
Creatinine (mg/dL)	2.4	(± 1.5)	1.6	(± 0.8)	0.139
Albumin (g/dL)	3.1	(± 0.6)	3.3	(± 0.6)	0.266
Na (mmol/L)	133	(± 4.3)	134	(± 3.6)	0.125
Lactate, mg/dL	33.9	(± 30.4)	23.2	(± 18.6)	0.086
LRINEC score > 8, *n* (%)	8	(61.5%)	8	(36.7%)	< 0.001^*^
Bacteremia, *n* (%)	9	(69.2%)	8	(36.4%)	0.001^*^
Hospital stay (days)	41.2	(± 16.9)	33.7	(± 21.1)	

Values are presented as mean ± standard deviation or number (%)

*PAD*, peripheral artery disease, *CKD* chronic kidney disease, *WBC* white blood cell, *CRP* C-reactive protein, *LRINEC* Laboratory Risk Indicator for Necrotizing Fasciitis

*p < 0.05

**Table 2:** Independent risk factors for mortality in late amputation NF using a multivariate analysis

Risk factor	OR	95% CI	*p* value
Hemorrhagic bullae	4.75	2.68–8.69	0.001^*^
Diabetes mellitus	1.62	1.15–7.14	0.15
PAD	3.20	1.12–10.58	0.001^*^
CRP	1.06	0.99–1.87	0.204
LRINEC score > 8	1.97	1.28–4.61	0.001^*^
Bacteremia	2.87	2.07–5.96	0.021^*^

*OR* odds ratio, *CI* confidence interval, *PAD* peripheral artery disease, *CRP* C-reactive protein, *LRINEC* Laboratory Risk Indicator for Necrotizing Fasciitis

*p < 0.05

Microbiology data regarding blood culture are shown in Table 3. In all, 159 patients (27.4%) were found to have positive isolated organisms. *Vibrio vulnificus* was the most frequent causative organism (34.6%). *Streptococcus* species and polymicrobial infections both accounted for 13.2% of infections. However, no difference between the amputation and non-amputation groups was found according to the isolated organisms.

**Table 3:** Microorganisms identified in blood culture in amputation and non-amputation groups with NF

Pathogens	Amputation group	Non-amputation group	Total, *n* (%)
	(*n* = 35)	(*n* = 124)	(*n* = 159)
MSSA	0 (0)	2 (1.3)	2 (1.3)
MRSA	4 (2.5)	6 (3.8)	10 (6.3)
*Streptococcus* species	4 (2.5)	17 (10.7)	21 (13.2)
*Escherichia coli*	1 (0.6)	2 (1.3)	3 (1.9)
*Pseudomonas aeruginosa*	7 (4.4)	3 (1.6)	10 (6.3)
*Vibrio vulnificus*	4 (2.5)	51 (32.1)	55 (34.6)
*Aeromonas* species	3 (1.6)	8 (5.0)	11 (6.9)
Other gram-positive	3 (1.6)	9 (5.7)	11 (6.9)
Other gram-negative	2 (1.3)	12 (7.5)	14 (8.8)
Polymicrobial	7 (4.4)	14 (8.8)	21 (13.2)

Microorganisms identified in blood culture did not show statistically significant differences between the two groups (*p >* 0.05). Values are shown as *n* (%)

*MSSA* methicillin-sensitive *Staphylococcus aureus*, *MRSA* methicillin-resistant *Staphylococcus aureus*

## Discussion

A number of studies have investigated risk factors associated with NF patient mortality, but only a limited amount of literature is available on the association between mortality and late amputation. In the mortality group, all 13 patients received fasciotomy and debridement at the first day of admission, 10 of them received multiple debridement and amputated at last and 3 of them amputated at second times of operation beyond 3 days after admission. Inadequate initial debridement for infection control, unexpectedly deteriorated clinical condition, and delay recognition may be the source of delay amputation, which led to mortality. Besides, few patients refused to receive primary amputation which caused delay amputation, which was also documented in the medical chart. Therefore, early recognition of high-risk NF patients is an important issue. Our findings suggest that an NF case should be treated early and aggressively and that primary amputation will prevent high-risk factors in patients that lead to mortality. Khamnuan et al. reported that clinical predictors for amputation in patients with NF included soft tissue swelling, skin necrosis, and gangrene [8]. Krieg et al. also reported that the presence of visible skin necrosis as an independent predictor of mortality emphasizes the outstanding importance of early diagnosis and prompt treatment to improve the prognosis [10]. Our study demonstrated that hemorrhagic bullae were highly associated with mortality in late amputation in NF, which is more precise than skin necrosis, also a late clinical manifestation and indicates an unfavorable progression of NF. Su et al. studied the LRINEC cut-off value associated with poor outcomes in 209 NF patients and demonstrated higher mortality and amputation rates in patients based on LRINEC scores [10]. The rates of early diagnosis (64 vs 70%), early operation (71 vs 70%), and time for operation (30 ± 51 vs 27.5 ± 51 min) were comparable between the two LRINEC groups. The overall mortality and amputation rates were 16% and 26%, respectively, whereas the rates of mortality (21% vs. 11%) and amputation (36% vs. 17%) in patients with LRINEC score ≥ 6 were higher than those who had LRINEC < 6 [11]. Wong et al. described the LRINEC that categorizes patients as “high risk” if the score is ≥ 8 [12]. El-Menyar et al. published results suggesting the LRINEC score might be useful for risk stratification and prognosis, in which cut-off value for predicting hospital mortality was 8 points in their study [13]. In our study, LRINEC score > 8 showed a statistically significant difference between mortality of the primary and late amputation groups. Chen et al. identified vascular disease as a common comorbidity of necrotizing soft tissue infections [14]. On the basis of this study, we found that peripheral artery disease was an important independent factor associated with mortality in late amputation. Chen et al. reported that the presence of *Streptococcus* group A in blood cultures was associated with a high risk of mortality in NF [15]. In our study, all NF patients underwent blood culture; the positivity rate was 27.4%. NF patients with bacteremia were more susceptible to death in the late amputation group.

Some individuals might be more susceptible to *V. vulnificus* infection following the consumption of contaminated seafood or following exposure of an open wound to seawater or marine animals, especially in situations where there is underlying chronic liver disease [16]. Most of our patients lived near the ocean and worked in fisheries, which is in support of the abovementioned findings*.* Tsai et al. reported that among patients with *Vibrio* NF, a systolic blood pressure of 90 mmHg, low platelet count, and a combination of hepatic dysfunction were associated with a higher mortality rate [17]. Although *Vibrio* NF has high mortality, our study showed that this had no clinical correlation with mortality in late amputation. Jabbour et al. reported that among gram-positive bacteria in NF, *Streptococcus* and *Staphylococcus* were the most commonly identified organisms [18]. In our study, *V. vulnificus* accounted for 34.6%, while *Streptococcus* species and polymicrobial infection both accounted for 13.2% of cases.

Lee et al. previously reported that early diagnosis and prompt treatment within 3 days post-injury or symptom onset should be the goal for treating patients with NF caused by *V*. *vulnificus* [9]. Wong et al. identified that delay in surgery for more than 24 h was correlated with increased mortality [1]. Hong et al. reported that early initiation of simple incision and drainage under regional anesthesia followed by complete debridement 24 h later is more feasible and effective for patients with *Vibrio* NF complicated by septic shock, as compared with the aggressive surgical debridement strategy [19]. Based on our findings, NF patients with hemorrhagic bullae, comorbidity with peripheral vascular disease, LRINEC score > 8, or the presence of bacteremia should receive early and primary amputation in order to prevent mortality. More studies are necessary to determine the benefit from surgical time and intervention for NF patients.

Most of the limitations of our study are related to its relatively small sample size. This results in a wide range in the confidence interval of our factors and also limits the evaluation of more factors.

## Conclusions

Emergency physicians are often the first to evaluate patients with NF and therefore should be aware of the risk and management of this disease. This study confirmed that peripheral artery disease, presence of hemorrhagic bullae, bacteremia, and LRINEC score > 8 are independent risk factors, which contribute to mortality in late amputation of NF. Patients with any of these predictors should be monitored closely and receive early aggressive treatment as primary amputation to prevent mortality. More studies are required to determine the significance of these findings and develop guidelines for management.

## Abbreviations

CI: Confidence interval
CKD: Chronic kidney disease
CRP: C-reactive protein
LRINEC: Laboratory Risk Indicator of Necrotizing Fasciitis
MRSA: Methicillin-resistant *Staphylococcus aureu*
MSSA: Methicillin-sensitive *Staphylococcus aureus*
NF: Necrotizing fasciitis
OR: Odds ratio
PAD: Peripheral artery disease
WBC: White blood cell

## References

[1] Wong C-H, Chang H-C, Pasupathy S, Khin L-W, Tan J-L, Low C-O (2003). Necrotizing fasciitis: clinical presentation, microbiology, and determinants of mortality. J Bone Joint Surg Am.

[2] Headley AJ (2003). Necrotizing soft tissue infections: a primary care review. Am Fam Physician.

[3] Ozalay M, Ozkoc G, Akpinar S, Hersekli MA, Tandogan RN (2006). Necrotizing soft-tissue infection of a limb: clinical presentation and factors related to mortality. Foot Ankle Int.

[4] Roje Z, Roje Z, Matić D, Librenjak D, Dokuzović S, Varvodić J (2011). Necrotizing fasciitis: literature review of contemporary strategies for diagnosing and management with three case reports: torso, abdominal wall, upper and lower limbs. World J Emerg Surg.

[5] Hakkarainen TW, Kopari NM, Pham TN, Evans HL (2014). Necrotizing soft tissue infections: review and current concepts in treatment, systems of care, and outcomes. Curr Probl Surg.

[6] Huang K-F, Hung M-H, Lin Y-S, Lu C-L, Liu C, Chen C-C (2011). Independent predictors of mortality for necrotizing fasciitis: a retrospective analysis in a single institution. J Trauma.

[7] Hsiao C-T, Weng H-H, Yuan Y-D, Chen C-T, Chen I-C (2008). Predictors of mortality in patients with necrotizing fasciitis. Am J Emerg Med.

[8] Khamnuan P, Chongruksut W, Jearwattanakanok K, Patumanond J, Tantraworasin A (2015). Necrotizing fasciitis: epidemiology and clinical predictors for amputation. Int J Gen Med.

[9] Lee YC, Hor LI, Chiu HY, Lee JW, Shieh SJ (2014). Prognostic factor of mortality and its clinical implications in patients with necrotizing fasciitis caused by *Vibrio vulnificus*. Eur J Clin Microbiol Infect Dis.

[10] Krieg A, Dizdar L, Verde PE, Knoefel WT (2014). Predictors of mortality for necrotizing soft-tissue infections: a retrospective analysis of 64 cases. Langenbeck's Arch Surg.

[11] Su YC, Chen HW, Hong YC (2008). Laboratory risk indicator for necrotizing fasciitis score and the outcomes. ANZ J Surg.

[12] Wong CH, Khin LW, Heng KS (2004). The LRINEC (Laboratory Risk Indicator for Necrotizing Fasciitis) score: a tool for distinguishing necrotizing fasciitis from other soft tissue infections. Crit Care Med.

[13] El-Menyar A, Asim M, Mudali IN (2017). The laboratory risk indicator for necrotizing fasciitis (LRINEC) scoring: the diagnostic and potential prognostic value. Scand J Trauma Resus Emerg Med.

[14] Chen KJ, Klingel M, McLeod S, Mindra S, Ng VK (2017). Presentation and outcomes of necrotizing soft tissue infections. Int J Gen Med.

[15] Chen IC, Li WC, Hong YC, Shie SS, Fann WC, Hsiao CT (2011). The microbiological profile and presence of bloodstream infection influence mortality rates in necrotizing fasciitis. Crit Care.

[16] Bross MH, Soch K, Morales R, Mitchell RB (2007). *Vibrio vulnificus* infection: diagnosis and treatment. Am Fam Physician.

[17] Tsai Y-H, Hsu RW-W, Huang K-C, Huang T-J (2010). Laboratory indicators for early detection and surgical treatment of vibrio necrotizing fasciitis. Clin Orthop Relat Res.

[18] Jabbour G, El-Menyar A, Peralta R, Shaikh N, Abdelrahman H, Mudali IN (2016). Pattern and predictors of mortality in necrotizing fasciitis patients in a single tertiary hospital. World J Emerg Surg.

[19] Hong GL, Dai XQ, Lu CJ, Liu JM, Zhao GJ, Wu B (2014). Temporizing surgical management improves outcome in Vibrio necrotizing fasciitis complicated with septic shock on admission. Burns.

